# Gaps in access and trust: essential medicine availability, affordability, and barriers to generic medicine use

**DOI:** 10.5588/pha.26.0033

**Published:** 2026-08-24

**Authors:** R. Sathiyamoorthy, P.R. Naik, J. Cherian, M.H. Rao, V. Dwarakanathan, V. A, P. Thekkur

**Affiliations:** 1 Department of Community and Family Medicine, All India Institute of Medical Sciences, Madurai, Tamil Nadu, India;; 2 Department of Community Medicine, Yenepoya Medical College, Mangaluru, India;; 3 Department of Community and Family Medicine, All India Institute of Medical Sciences, Madurai, Tamil Nadu, India;; 4 All India Institute of Medical Sciences, Madurai, Tamil Nadu, India;; 5 Department of Community Medicine, Employees State Insurance Corporation Medical College, Chennai, Tamil Nadu, India;; 6 Department of Community Medicine, Pondicherry Institute of Medical Sciences, Puducherry, India;; 7 Centre for Operational Research, International Union Against Tuberculosis and Lung Disease, Paris, France;; 8 UNICEF, UNDP, World Bank, WHO Special Programme for Research and Training in Tropical Diseases (TDR), Geneva, Switzerland.

**Keywords:** Tamil Nadu, India, universal health coverage, supply chain management, drug safety, operational research, SORT IT

## Abstract

**SETTING:**

Selected public health facility pharmacies, private pharmacies, and Jan Aushadhi Kendras (JAKs) outlets dispensing generic medicines in four districts of Tamil Nadu, India.

**OBJECTIVE:**

To compare essential medicine availability, affordability, and knowledge on generic medicines among clients across pharmacy types. Additionally, to explore barriers to the use of generic medicines.

**DESIGN:**

A concurrent mixed-methods study with a quantitative cross-sectional assessment of the availability of 50 essential medicines in 70 pharmacies and structured interview of 1,750 clients. Eight focus group discussions with clients and 24 in-depth interviews with health care providers to qualitatively explore barriers.

**RESULTS:**

The median number of essential medicines available in private pharmacies (43) was higher than that in public pharmacies (30) and JAKs (29) (*P* < 0.001). The medicine purchase was unaffordable (expenditure >6USD) for 92% of clients in JAKs and 98% of private pharmacies. Knowledge of generic medicine was low among clients attending public (24%) and private (31%) pharmacies. Barriers to generic medicine use included unavailability of medicines, concerns about effectiveness, and suboptimal marketing.

**CONCLUSION:**

JAKs have to address the unavailability of medicines, high unaffordability, and barriers to utilisation by expanding their drug baskets, further reducing costs, and implementing strategies to build trust in generic medicines.

## Graphical abstract

**Figure:**
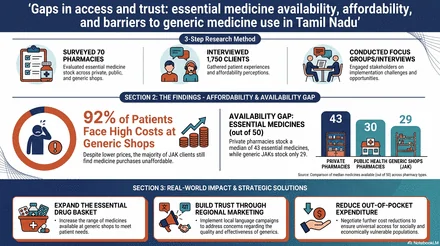

Universal access to safe, affordable, effective, and quality essential medicines is a key target of the Sustainable Development Goals (SDGs).^1,2^ To achieve universal health coverage (UHC), it is imperative to ensure accessibility and affordability of medicines by reducing out-of-pocket expenditure (OOPE). In India, it is estimated that OOPE on medicine alone pushed 38 million persons into poverty during 1994–2014.^3^ In 2021–2022, an estimated OOPE on health was 45 billion USD, of which 43% was spent only on medicines.^4^ The National Pharmaceutical Pricing Policy (2012) was introduced to control drug-formulation prices and limit market-based pricing of essential medicines.^5^ Additionally, Jan Aushadhi Yojana (2015) was launched to improve the availability and affordability of essential medicines though dispensing generic formulations through Jan Aushadhi Kendra (JAK) outlets.^6^ Currently, pharmacies in public health facilities dispense drugs free of cost or with a nominal user fee, private pharmacies dispense brand-name drugs with OOPE, and JAKs dispense generic drugs at a low cost.

Globally, generic medicines are recognised as a cost-effective alternative to brand-name drugs.^7^ Generic drugs contain the same active ingredients and therapeutic effect, but with 30%–80% less cost than their brand-name counterparts.^8^ Despite cost advantages and clinical equivalence, uptake of generic medicines is poor as consumers harbour doubts about the effectiveness and safety of the drugs as they are available at a lower price.^9^ About 64% of doctors in Tamil Nadu state (2022) believed generic medicines are effective and safe as brand-name drugs, but more than 50% were reluctant to prescribe them in critical or life-threatening conditions. Despite policies promoting generic medicines, lingering distrust and information gaps among clients and health care providers could lead to suboptimal utilisation of JAKs.^10^ The availability, affordability, and perceptions of clients determine the use of generic medicines and the utilisation of JAKs. Thus, it is imperative for policymakers to understand these aspects to make evidence-based decisions for improving the utilisation of JAKs. Such a comprehensive assessment would require a mixed-methods approach, combining quantitative and qualitative exploration.^11^

Thus, we conducted a study in four districts of Tamil Nadu, India, to compare across public health facility pharmacies, private pharmacies, and JAKs: 1) the median number of available essential medicines, 2) the proportion of clients who could afford the medicines purchased, and 3) the knowledge, attitude, and practice (KAP) related to generic medicines. Additionally, we explored the acceptability of generic medicines as perceived by doctors, pharmacists, facility administrators, and clients.

## METHODS

This was a concurrent mixed-methods study.^11^ The quantitative component included a checklist-based cross-sectional assessment of pharmacies and a cross-sectional client survey. The qualitative component was a descriptive study with focus group discussions (FGDs) and in-depth interviews (IDIs).

### General Setting

This study was conducted in four purposively selected districts of the state of Tamil Nadu, India. Tamil Nadu is the sixth largest state in southern India with a population of about 72 million. The following districts were selected for the study: 1) Chennai district is a metropolitan city and has an urban population, 2) Madurai district has a tier-two city with both urban and peri-urban population, 3) Villupuram district has a semi-urban population, and 4) Ramanathapuram district has a predominantly rural population. This selection helped to capture the varied spectrum of geographic, socio-economic, and health care access contexts.

### Public health facility pharmacies

These are drug dispensing outlets functioning within government-run health facilities like primary health centres, community health centres, and district hospitals. These dispense medicines prescribed by the treating physician, often at no cost or at subsidised rates. These outlets are crucial for economically disadvantaged populations accessing care at public health facilities. Tamil Nadu has around 1,700 public health facility pharmacies.

### Private pharmacies

These are operated under market-based mechanisms and are typically attached to private hospitals or run as independent retail outlets. These establishments predominantly stock and dispense brand-name drugs, which are priced higher than generic equivalents. Clients can even procure medicines over the counter without a prescription.

### Jan Aushadhi Kendras

These are generic medicine outlets established under the Pradhan Mantri Bhartiya Jan Aushadhi Pariyojana, implemented by the Pharmaceuticals and Medical Devices Bureau of India. JAKs dispense quality-assured generic formulations at significantly lower prices than brand-name drugs, ideally for those with a prescription. There are about 1,584 JAKs functional in Tamil Nadu state.^12^

### Study population

To assess the availability of essential medicines, we included six public health facility pharmacies, six private pharmacies, and six JAKs from each study district. The selection of facilities was guided by the WHO/Health Action International (WHO/HAI) methodology.^13^ In each district, the district hospital was selected first. The five other public health facilities, which were at least an hour’s drive apart, were included. We included a private pharmacy and a JAK within 10 km of every public health facility pharmacy in the study. To assess the affordability of medicines and KAP towards generic medicines, 25 adult clients (aged ≥18 years) purchasing medicines from each of the above selected pharmacies during October 2023 to January 2024 were included. About 600 clients purchasing from each pharmacy type were included (a total of 1800 clients). To explore the acceptability of generic medicines, two FGDs were conducted with clients and six IDIs with health care providers (doctors, pharmacists, and facility administrators) in each study district. In total, eight FGDs and 24 IDIs were conducted. Those individuals who were vocal and informed about the functioning of pharmacies were purposively selected for both FGDs and IDIs. In each FGD, there were 8–10 participants. Of the 24 IDI participants, six were females.

### Data variables, source, and collection

In every pharmacy, data on the availability of each of the 50 essential medicines were assessed using a structured checklist. The 50 essential medicines were derived from the National List of Essential Medicines using the WHO/HAI methodology, and they included all 14 medicines mentioned in the WHO global core list.^13^ Five trained field investigators assessed availability by direct observation of stock registers and physical verification of inventory. A structured questionnaire was used to capture the affordability of medicines and KAP towards generic medicines (Supplementary Data Table S2). Information on socio-demographic characteristics (age, sex, marital status, occupation, education, and monthly family income), prescription source and list, and the cost of medicines purchased was captured. Two questions on knowledge, one on practice (binary answer: yes/no), and four on attitude (Likert scale: strongly disagree/disagree/neither disagree nor agree/agree/strongly agree) were included to assess KAP regarding generic medicines. Trained data collectors conducted exit interviews of 25 clients in each of the selected pharmacies. Data were captured directly into Epicollect5, a mobile-based data collection platform, to ensure completeness and real-time validation.

Pre-tested discussion guides tailored to each stakeholder group were used for FGDs and IDIs (Annexures 3 and 4). All interviews were conducted in the local language (Tamil) by the investigator (male, medical doctor) and trained research staff. The interviews were conducted in either a health facility or a nearby community space preferred by the interviewee. In addition to the audio recording, field investigators maintained detailed field notes to capture non-verbal cues and contextual observations. The average duration of an FGD was 42 min and IDI was 18 min.

### Operational definition

Affordability: The purchase of medicine was considered affordable if the cost of medicine was less than or equal to one day’s wage of the lowest-paid unskilled government worker (INR 403/day) in Tamil Nadu.^14,15^

Favourable attitude: Clients’ responses of ‘agree’ or ‘strongly agree’ to the attitude questions were considered a favourable attitude.

### Data entry and analysis

All statistical analyses were performed using Stata version 14 (StataCorp LP, College Station, TX, USA). The number of essential medicines across pharmacy types was summarised using median (interquartile range [IQR]) and compared using the Kruskal–Wallis test. For each of the 14 WHO core essential medicines, the number and proportion of pharmacies that had it were calculated and compared across pharmacy types using the χ² test. Clients’ socio-demographic characteristics, prescription source, affordability, and KAP questions were summarised using frequencies and percentages, and compared across pharmacy types using the χ² test. Statistical significance was determined at a 5% level (*P* value < 0.05).

Audio recordings of FGDs and IDIs were transcribed verbatim in the local language (Tamil) and translated into English. Manual content analysis was conducted with inductive coding of transcripts. Two researchers conducted coding independently using QDA Miner Lite to maintain analytical rigour. Any discrepancies in code assignment or theme development were discussed and resolved through consensus. The findings were reported as per the ‘Consolidated Criteria for Reporting Qualitative Research’ guidelines.^16^

### Ethical statement

Ethical and administrative approvals were obtained from the Jawaharlal Institute of Postgraduate Medical Education and Research (JIPMER) Institutional Ethics Committee, Puducherry (JIP/IEC-OS/438/2023), and the Directorate of Public Health and Preventive Medicine, respectively. Written informed consent was obtained from all participants.

## RESULTS

In total, 70 pharmacies were included, three JAKs rejected participation, and one additional private pharmacy was included. Of the 70 pharmacies, the median (IQR) number of essential medicines (out of 50) available was 33 (28–41). However, it was 30 (28–33) in public health facility pharmacies, 43 (34–45) in 25 private pharmacies, and 29 (22-39) in 21 JAKs, respectively (*P* < 0.001). Dunn’s post-hoc test with Bonferroni correction showed significantly higher availability in private pharmacies compared with public facilities (*P* < 0.001) and JAKs (*P* < 0.001). However, there was no difference between public facilities and JAKs (*P* = 0.366). The antibiotics (19%–81%), paracetamol suspension (19%), and diazepam (43%) were rarely available in JAKs (Table 1).

**TABLE 1.** Availability of WHO Global core essential medicines (by disease categories) at public health facility pharmacies, private pharmacies, and Jan Aushadhi Kendras in four selected districts of Tamil Nadu, India, 2023–2024.

Disease categories and WHO global core essential medicines	Public, *N* = 24		Private, *N* = 25		Jan Aushadhi Kendras, *N* = 21		*P* value^B^
	n	%^A^	n	%^A^	n	%^A^	
Asthma							
Salbutamol inhaler 100 mcg/dose	0	0	18	72	9	43	<0.001
Diabetes							
Metformin 500 mg cap/tab	24	100	25	100	18	86	0.03
Cardiovascular diseases							
Bisoprolol 5 mg cap/tab	3	13	16	64	16	76	<0.001
Captopril 25 mg cap/tab	22	92	23	92	20	95	0.88
Simvastatin 20 mg cap/tab	3	13	23	92	16	76	<0.001
Depression							
Amitriptyline 25 mg cap/tab	17	71	14	56	16	76	0.31
Infectious diseases							
Ciprofloxacin 500 mg cap/tab	24	100	25	100	8	38	<0.001
Co-trimaxazole suspension 8 + 40 mg/ml	11	46	21	84	13	62	0.02
Amoxicillin 500 mg cap/tab	0	0	25	100	17	81	<0.001
Ceftriazone 1 g/vial	11	46	15	60	4	19	0.02
Central nervous system							
Diazepam 5 mg cap/tab	24	100	13	52	9	43	<0.001
Pain/inflammation							
Diclofenac 50 mg cap/tab	21	88	20	80	17	81	0.76
Paracetamol suspension 24 mg/ml	14	58	19	76	4	19	<0.001
Ulcer							
Omeprazole 20 mg cap/tab	24	100	25	100	12	57	<0.001

A Column percentage.

B χ² test.

### Socio-demographic characteristics of end users

In total, 1,750 clients were included, with 603 from public health facility pharmacies, 599 from private pharmacies, and 548 from JAKs. The demographic characterises of clients varied across type of pharmacy. Those attending JAKs were mostly elderly (27%) and males (70%), whereas women (64%), unemployed (44%), illiterates (26%), and those with lower monthly family income (15,000 [10,000–25000] INR) frequented public health facility pharmacy (Table 2).

**TABLE 2.** Socio-demographic characteristics of clients, amount spent on purchase of medicines, and affordability across public health facility pharmacies, private pharmacies, and Jan Aushadhi Kendras (JAKs) in selected districts of Tamil Nadu, India 2023–2024.

Characteristics^A^	Public		Private		JAK		*P* value^B^
	n	%^C^	n	%^C^	n	%^C^	
Total^C^	603	100	599	100	548	100	
Age							
18–59	487	81	494	83	402	73	<0.001
60 and above	116	19	105	17	146	27	
Gender							
Men	215	36	402	67	385	70	<0.001
Women	388	64	197	33	163	30	
Marital status							
Married	525	87	497	83	465	85	<0.001
Unmarried	33	6	79	13	57	10	
Widowed and others	45	7	23	4	26	5	
Occupation							
Private sector	171	28	198	33	187	34	<0.001
Government sector	27	5	28	5	35	6	
Self employed	139	23	197	33	148	27	
Unemployed	266	44	176	29	178	33	
Education							
Illiterate	156	26	95	15	79	15	<0.001
Primary and middle school	147	24	99	17	50	9	
Secondary and higher secondary	183	30	148	25	199	36	
Graduate and above	117	20	257	43	220	40	
Median (IQR) monthly family income	15,000	10,000–25,000	35,000	25,000–50,000	27,500	20,000–40,000	<0.001
Median (IQR) amount spent per month towards purchasing medicines	200	0–600	2000	1,000–3,000	1,200	500–2,500	<0.001
Affordability^D^							
Affordable	338	56	10	2	41	8	<0.001
Not affordable	265	44	589	98	507	92	
Prescription							
Not available	5	1	134	23	69	13	
Private hospital	7	1	331	55	252	46	<0.001
Public hospital	587	97	14	2	98	18	
Nursing homes	0	0	5	1	18	3	
General practitioners and others	4	1	115	19	111	20	

A Column percentage.

B χ² test.

C Row percentage.

D Medicines were affordable if they cost less than or equal to a day’s wage, and unaffordable if they exceeded it. The lowest-paid unskilled government worker in Tamil Nadu earned INR 403 a day.

The median (IQR) amount (in INR) spent by clients on monthly basis for purchasing medicines was 200 (0–600) in public health facility pharmacies, 2,000 (1,000–3,000) in private pharmacies, and 1,200 (500–2,500) in JAKs. The purchase of drugs was affordable for 56% clients in public health facility pharmacies, 2% in private pharmacies, and 8% in JAKs.

### Knowledge, attitude, and use of generic medicines

Only 24% of clients of public health facility pharmacies and 31% in private pharmacies were aware of the term generic medicine, whereas it was 69% in JAKs. Among those aware of generic medicines, correct understanding of generic medicine was found in 71%, 70%, and 91% of public, private, and JAK clients, respectively. In clients who were aware of generic medicine, the favourable attitude towards it was relatively lower in clients of private pharmacy (43%–60%) and public health facility pharmacies (87%–96%) compared to those of JAKs (96%–98%). Only 73% clients of private pharmacies ever used generic medicine compared to 96% in public health facility pharmacies and JAKs (Table 3).

**TABLE 3.** Knowledge, attitude, and practice related to generic medicines among end users across public health facility pharmacies, private pharmacies, and Jan Aushadhi Kendras (JAKs) in selected districts of Tamil Nadu, India 2023-2024.

Component	Public		Private		JAK		*P* value^A^
	n	%^B^	n	%^B^	n	%^B^	
Total	603	100	599	100	548	100	
Knowledge (n = 1750)							
Aware of the term generic medicine	147	24	186	31	376	69	<0.001
Correct understanding of what constitutes generic medicine (n = 709)	105	71	131	70	343	91	<0.001
Attitude (n = 708)							
Is generic medicine being of same quality as branded medicine?	128	87	96	52	363	97	<0.001
Is generic medicine work as effectively as branded medicine?	129	88	80	43	361	96	<0.001
Is generic medicine being as safe as branded medicine?	137	93	111	60	369	98	<0.001
If I were ill, I would be happy to take a generic medicine if my doctor prescribed it for me	141	96	99	53	362	97	<0.001
Practice (n = 708)							
History of having used generic medicines in the past	141	96	135	73	361	96	<0.001

A χ² test.

B Column percentage.

### Qualitative results

In total, 44 unique codes were deduced directly from the transcripts and were grouped into 21 emergent sub-themes, which were then synthesised into four overarching themes related to barriers to using generic medicines (see Figure and Supplementary Data Table S1). The themes and suggested solutions are described below.

**FIGURE.**
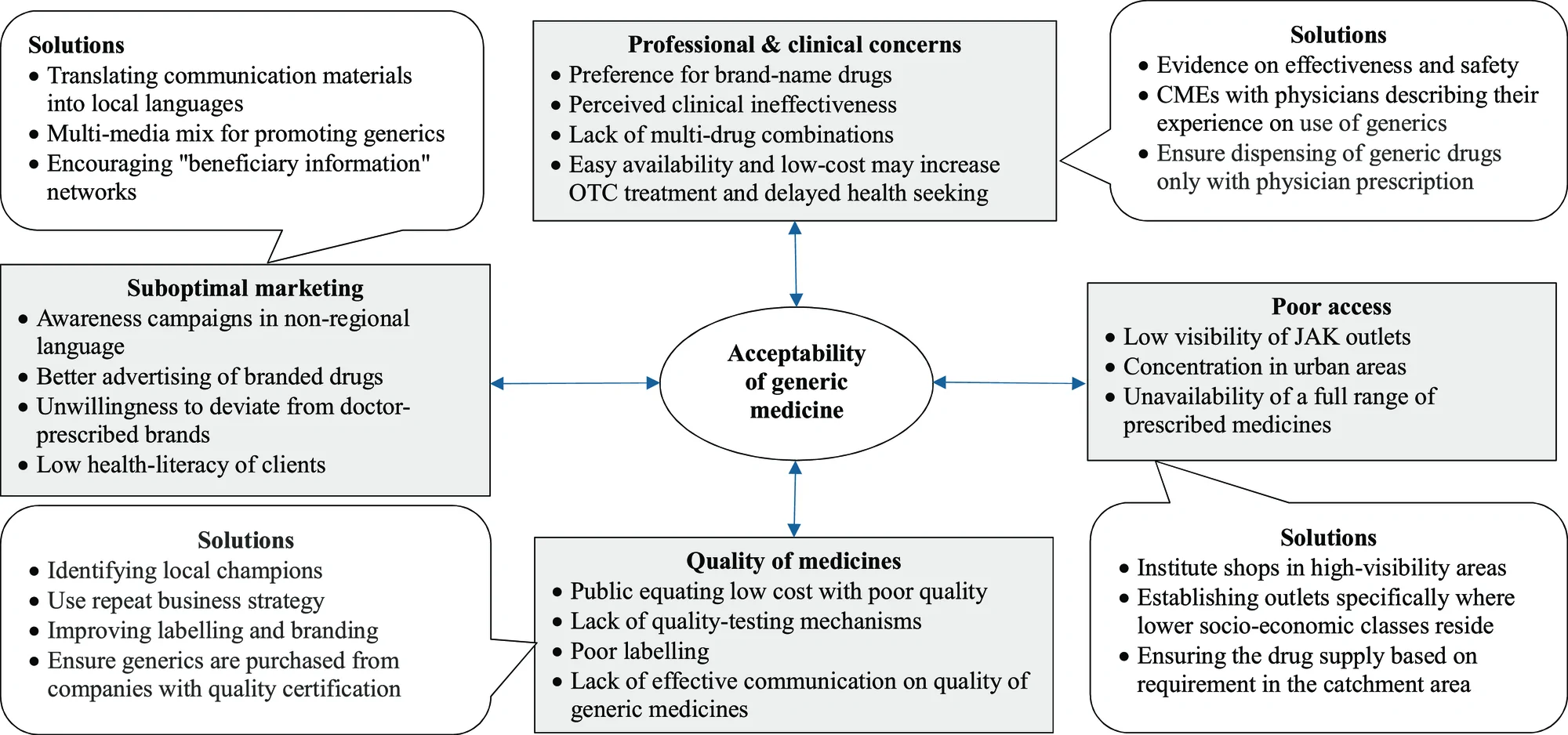
Thematic diagram depicting barriers to acceptability of generic medicines as perceived by the doctors, pharmacists, facility administrators, and end users in selected districts of Tamil Nadu, India. OTC = over-the-counter; CME = continuing medical education; JAK = Jan Aushadhi Kendra.

### Professional and clinical concerns of physicians

Health care providers said generic medicines weren’t effective, so they switched to brand-name drugs. They worried about the lack of multi-drug combinations in generics, which caused ‘pill burden’. They also feared that the easy availability and low cost of generics would encourage people to buy medicines without a prescription, which could lead to delayed health-seeking and inappropriate drug use, including antibiotics. To address these issues, they suggested generating evidence on the effectiveness and safety of generics, providing continuing medical education on successful generic medicine use, and ensuring that JAKs only dispense medicines with a doctor’s prescription.

### Poor access to medicines in JAKs

Both health care workers and clients felt the visibility of JAKs was low, as the outlets were mostly located deep inside the streets, without signage or advertisements. As JAKs are present mostly in urban areas, people from rural areas have poor access to generic drugs. Clients expressed that the full range of prescribed drugs was not available in JAKs, requiring them to purchase from private pharmacies. The solutions proposed by health care providers and clients included establishing outlets in high-visibility locations with better access for people from rural areas and expanding the medicine basket in the JAKs.

### Concerns about the quality of medicines

Clients perceived that the low cost of generic medicines indicated poor quality. Clients also mentioned that their perception was justified by poor labelling of generic medicines and the lack of information on drug quality in JAKs. The solutions proposed by clients include identifying ‘local champions’ to speak on the quality of generic medicine and improving labelling and branding. However, health care providers recommended that JAKs procure drugs only from reliable companies with quality certifications and adopt a repeat business strategy to enhance clients’ trust in the quality of generic medicines.

### Suboptimal marketing of generic medicines

Health care providers and clients reported that government campaigns promoting generic medicines were ineffective in reaching the general public because they were not in regional languages. In contrast, brand-name drugs are better advertised. Clients also mentioned that they were hesitant to deviate from doctor-prescribed brand-name drugs. Solutions proposed include employing a multimedia mix and social marketing to promote generic medicines in regional languages, creating peer-support groups of satisfied clients, and engaging physicians to prescribe generic medicines.

Qualitative findings helped to understand several quantitative patterns. Lower JAK utilisation reflects structural barriers such as poor signage and hidden placement that filter out rural, less-literate populations, as stakeholders described. The specific shortfalls in antibiotics and paediatric formulations, captured quantitatively, were reiterated during qualitative exploration. Non-availability of drugs forced clients to use unaffordable private pharmacies, compounding OOPE despite the intended financial protection.

## DISCUSSION

This study comprehensively assessed the availability of essential medicines, affordability, and perceptions on generic medicines across pharmacy types in a state in India. The study has five key findings. First, the availability of essential medicines was lower in JAKs than in private pharmacies, with more than half of JAKs having fewer than 30 of the 50 essential medicines. Second, the clients accessing JAKs and public health facility pharmacies are mostly socially and economically vulnerable. Third, purchasing medicines exceeded the WHO/HAI affordability threshold for a large proportion of clients, including those accessing JAKs. Fourth, the majority of clients in non-JAK pharmacies were unaware of generic medicines and rarely used them. Fifth, health care providers and clients perceived barriers on the safety, access, quality, and marketing strategies for generic medicines with potential solutions to overcome them.

The United Nations General Assembly Political Declaration on UHC (2019) called member states to promote equitable access and affordability of essential medicines, including generics.^17^ The SDG and WHO’s access programme recommend use of generics and pricing policies to achieve this.^18^ Thus, this study provides evidence from India on access to essential medicines, affordability, and clients’ perception of generic medicines.

Similar to our study findings, a study from Maharashtra, India, reported a poor availability of essential medicines in JAKs.^14^ During the qualitative exploration, clients and health care providers also highlighted the unavailability of all prescribed medicines in JAKs, necessitating the purchase of drugs from private pharmacies. The private pharmacies had almost all the essential medicines and could mostly dispense all the prescribed drugs, as reported elsewhere.^19–24^ Thus, as suggested by clients and health care providers, the JAK drug basket has to be expanded. High availability of diazepam in public facilities reflects its inclusion in essential medicine lists and use in emergency and inpatient care, despite regulatory controls on benzodiazepines. The Pharmaceutical and Medical Devices Bureau of India (PMBI) should procure from both public and private manufacturers to address missing essential medicines and fast-track the procurement of high-need but low-competition generic molecules, such as paediatric formulations (paracetamol suspension) and antibiotics. Additionally, similar efforts to improve the availability of essential medicines in public health facility pharmacies need to be prioritised, given that availability has remained low.

Previous studies have also reported that the cost of JAK medicines was lower than that of brand-name drugs dispensed through private pharmacies.^14,19,23,25,26^ During qualitative exploration, health care providers and clients also acknowledged and appreciated the low cost of generic medicines in JAKs. Despite this, as shown in other studies, most clients found purchasing drugs from JAKs unaffordable.^23,24,26^ However, PMBI can further reduce medicine costs by pooling multi-year tenders to negotiate higher discounts with manufacturers, leveraging JAKs’ national scale. Previous studies have also reported poor knowledge, an unfavourable attitude, and low use of generic medicines, consistent with our study findings.^27,28^ On qualitative exploration, suboptimal marketing of generic medicines was found to be a potential reason for poor knowledge and attitude. As suggested by clients and health care providers, demand-generation activities through mass-media campaigns in regional languages and the engagement of treating physicians to promote the use of generic medicines have to be prioritised. Additionally, poor access to JAKs and concerns about quality of generic medicines were reported as the major barriers for uptake of generic drugs. Similar concerns have been reported from other study settings.^9,29,30^ As suggested by clients and health care providers, there is a need for improving the visibility of JAK outlets. To address concerns about quality, the labelling and branding of generic medicines need to be improved, and the medicines should be procured only from manufacturers that adhere to WHO Good Manufacturing Practice. There is also a need for periodic independent assessment of the quality and effectiveness of generic medicines dispensed in JAKs.

The strengths of the study included independent assessment by trained research staff in a programmatic setting, use of standard WHO/HAI methodology, a large sample size, and a mixed-methods design to triangulate findings with perceptions from health care providers and clients. The limitations include the selection bias due to non-participation of some JAKs, potentially overestimating availability. Additionally, inclusion of only current users may result in self-selection and social desirability bias. We failed to capture potential variation in drug purchase and costing across acute and chronic conditions.

## CONCLUSIONS

JAKs have limited access to essential medicines. Although generic medicines in JAKs are cheaper than brand-name drugs in private pharmacies, they remain unaffordable for the majority of clients. Clients who access non-JAK pharmacies are unaware of generic medicines, largely due to poor marketing and limited access. Thus, there is an urgent need to improve the availability of essential medicines in JAKs, further reduce the cost of generic medicines dispensed through JAKs, and implement communication strategies to increase demand for generic medicines.
